# Poly[diaqua­bis­(μ_3_-1*H*-imidazole-4,5-dicarboxyl­ato)(μ_2_-sulfato)­diytterbium(III)]

**DOI:** 10.1107/S1600536811045673

**Published:** 2011-11-05

**Authors:** Li-Cai Zhu

**Affiliations:** a School of Chemistry and Environment, South China Normal University, Guangzhou 510631, People’s Republic of China

## Abstract

In the title compound, [Yb_2_(C_5_H_2_N_2_O_4_)_2_(SO_4_)(H_2_O)_2_]_*n*_, the Yb^III^ ion is eight-coordinated by four O atoms and one N atom from three imidazole-4,5-dicarboxyl­ate ligands, two O atoms from one SO_4_^2−^ anion  (site symmetry 2), as well as one O atom of a water mol­ecule, giving a bicapped trigonal–prismatic coordination geometry. The metal coordination units are connected by bridging imidazole-4,5-dicarboxyl­ate and sulfate ligands, generating a heterometallic layer. The layers are stacked along the *a* axis *via* N—H⋯O, O—H⋯O, and C—H⋯O hydrogen-bonding inter­actions, generating a three-dimensional framework.

## Related literature

For the application of multifunctional organic ligands containing O- and N-donors in the design of metal-organic frameworks, see: Cheng *et al.* (2006 ▶); Kuang *et al.* (2007 ▶); Sun *et al.* (2006 ▶); Zhu *et al.* (2010 ▶).
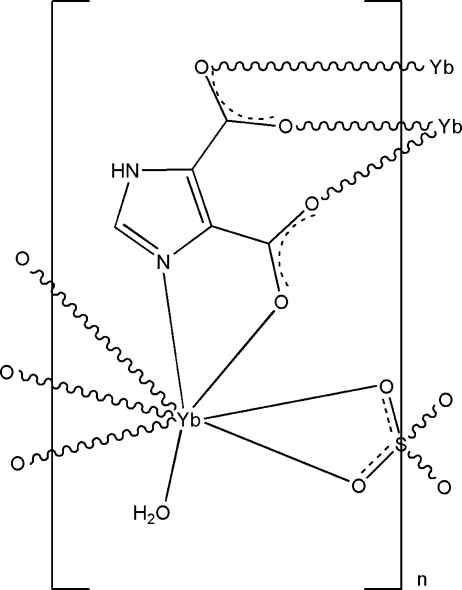


## Experimental

### 

#### Crystal data

[Yb_2_(C_5_H_2_N_2_O_4_)_2_(SO_4_)(H_2_O)_2_]
*M* *_r_* = 786.35
Monoclinic, 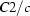

*a* = 21.1089 (14) Å
*b* = 6.5584 (4) Å
*c* = 12.8766 (9) Å
β = 105.874 (1)°
*V* = 1714.7 (2) Å^3^
*Z* = 4
Mo *K*α radiation
μ = 11.05 mm^−1^
*T* = 296 K
0.20 × 0.18 × 0.15 mm

#### Data collection

Bruker APEXII area-detector diffractometer
Absorption correction: multi-scan (*SADABS*; Sheldrick, 1996 ▶) *T*_min_ = 0.126, *T*_max_ = 0.191
4239 measured reflections
1534 independent reflections
1392 reflections with *I* > 2σ(*I*)
*R*_int_ = 0.023

#### Refinement

*R*[*F* ^2^ > 2σ(*F* ^2^)] = 0.020
*wR*(*F* ^2^) = 0.047
*S* = 1.09
1534 reflections
150 parameters
4 restraints
H atoms treated by a mixture of independent and constrained refinement
Δρ_max_ = 0.66 e Å^−3^
Δρ_min_ = −0.99 e Å^−3^

### 

Data collection: *APEX2* (Bruker, 2004 ▶); cell refinement: *SAINT* (Bruker, 2004 ▶); data reduction: *SAINT*; program(s) used to solve structure: *SHELXS97* (Sheldrick, 2008 ▶); program(s) used to refine structure: *SHELXL97* (Sheldrick, 2008 ▶); molecular graphics: *SHELXTL* (Sheldrick, 2008 ▶); software used to prepare material for publication: *SHELXL97*.

## Supplementary Material

Crystal structure: contains datablock(s) I, global. DOI: 10.1107/S1600536811045673/pv2472sup1.cif

Structure factors: contains datablock(s) I. DOI: 10.1107/S1600536811045673/pv2472Isup2.hkl

Additional supplementary materials:  crystallographic information; 3D view; checkCIF report

## Figures and Tables

**Table 1:** Hydrogen-bond geometry (Å, °)

*D*—H⋯*A*	*D*—H	H⋯*A*	*D*⋯*A*	*D*—H⋯*A*
N2—H1⋯O6^i^	0.87 (5)	2.09 (3)	2.925 (6)	161 (5)
O1*W*—H2*W*⋯O2^ii^	0.82 (2)	1.94 (3)	2.693 (5)	151 (5)
O1*W*—H1*W*⋯O3^iii^	0.82 (6)	2.24 (4)	2.896 (5)	138 (5)
O1*W*—H1*W*⋯O4^iii^	0.82 (6)	2.51 (6)	3.308 (5)	167 (5)
C5—H5⋯O5^iv^	0.93	2.52	3.347 (6)	149

Symmetry codes: (i) 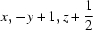; (ii) 
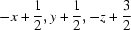
; (iii) 
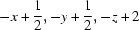
; (iv) 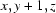.

**Fig. 1.**
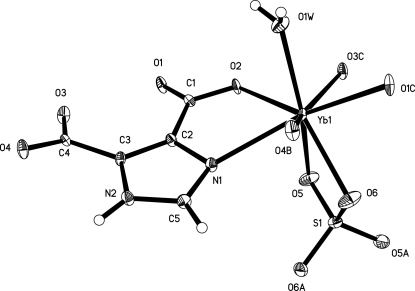
The molecular structure of the title comples showing atomic-numbering scheme and displacement ellipsoids drawn at 30% probability level. Symmetry codes: A = 1 - x, y, 1.5 - z; B = x, 1 - y, -1/2 + z; C = x, -y, -1/2 + z.

**Fig. 2.**
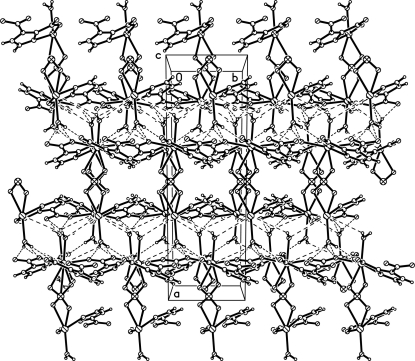
A view of the three-dimensional structure of the title compound, the hydrogen bonding interactions have been drawn as broken lines.

## supplementary crystallographic information

### Comment

In the past few years, the application of multifunctional organic ligands containing O– and N–donors to design metal-organic frameworks are of increasing interest, not only because of their impressive topological structures, but also due to their versatile applications in ion exchange, magnetism, bimetallic catalysis and luminescent probe (Cheng *et al.*, 2006; Kuang *et al.*, 2007; Sun *et al.*, 2006; Zhu *et al.*, 2010). As an extension of this research, the structure of the title compound, a new metal-organic framework, has been determined which is presented in this artcle.

The asymmetric unite of the title compound (Fig. 1), contains a Yb^III^ ion, an imidazole-4,5-dicarboxylate ligand, a half SO_4_^2-^ anion, and a coordinated water molecule. The Yb^III^ ion is eight-coordinated by four O atoms and a N atom from three imidazole-4,5-dicarboxylate ligands, two O atoms from a SO_4_^2-^ anion as well as a coordinated water molecule, giving a bicapped trigonal prismatic coordination geometry. The metal coordination units are connected by bridging imidazole-4,5-dicarboxylate and sulfate ligands, generating a two-dimensional heterometallic layer. The two-dimensional layers are stacked along *a* axis *via* N—H···O, O—H···O, and C—H···O hydrogen-bonding interactions to generate the three-dimensional framework (Table 1 and Fig. 2).

### Experimental

A mixture of Yb_2_O_3_ (0.099 g, 0.25 mmol), imidazole-4,5-dicarboxylic acid (0.156 g, 1 mmol), and H_2_O (7 ml) was sealed in a 20 ml Teflon-lined reaction vessel at 443 K for 5 days then slowly cooled to room temperature. The product was collected by filtration, washed with water and air-dried. Colorless block crystals suitable for X-ray analysis were obtained.

### Refinement

H atoms bonded to C atoms were positioned geometrically and refined as riding, with C—H = 0.93 Å and *U*_iso_(H) = 1.2 *U*_eq_(C). H atoms bonded to N atoms water molecules were found from difference Fourier maps and refined isotropically with a restraint of N—H = 0.87 Å, O—H = 0.82 Å and *U*_iso_(H) = 1.5 *U*_eq_(N, O).

### Crystal data

**Table:**

[Yb_2_(C_5_H_2_N_2_O_4_)_2_(SO_4_)(H_2_O)_2_]	*F*(000) = 1456
*M**_r_* = 786.35	*D*_x_ = 3.046 Mg m^−^^3^
Monoclinic, *C*2/*c*	Mo *K*α radiation, λ = 0.71073 Å
Hall symbol: -C 2yc	Cell parameters from 2574 reflections
*a* = 21.1089 (14) Å	θ = 3.3–28.0°
*b* = 6.5584 (4) Å	µ = 11.05 mm^−^^1^
*c* = 12.8766 (9) Å	*T* = 296 K
β = 105.874 (1)°	Block, colorless
*V* = 1714.7 (2) Å^3^	0.20 × 0.18 × 0.15 mm
*Z* = 4	

### Data collection

**Table:**

Bruker APEXII area-detector diffractometer	1534 independent reflections
Radiation source: fine-focus sealed tube	1392 reflections with *I* > 2σ(*I*)
graphite	*R*_int_ = 0.023
φ and ω scan	θ_max_ = 25.2°, θ_min_ = 2.0°
Absorption correction: multi-scan (*SADABS*; Sheldrick, 1996)	*h* = −25→22
*T*_min_ = 0.126, *T*_max_ = 0.191	*k* = −7→7
4239 measured reflections	*l* = −14→15

### Refinement

**Table:**

Refinement on *F*^2^	Primary atom site location: structure-invariant direct methods
Least-squares matrix: full	Secondary atom site location: difference Fourier map
*R*[*F*^2^ > 2σ(*F*^2^)] = 0.020	Hydrogen site location: inferred from neighbouring sites
*wR*(*F*^2^) = 0.047	H atoms treated by a mixture of independent and constrained refinement
*S* = 1.09	*w* = 1/[σ^2^(*F*_o_^2^) + (0.0216*P*)^2^ + 5.6648*P*] where *P* = (*F*_o_^2^ + 2*F*_c_^2^)/3
1534 reflections	(Δ/σ)_max_ = 0.001
150 parameters	Δρ_max_ = 0.66 e Å^−^^3^
4 restraints	Δρ_min_ = −0.99 e Å^−^^3^

### Special details

**Table:**

Geometry. All e.s.d.'s (except the e.s.d. in the dihedral angle between two l.s. planes) are estimated using the full covariance matrix. The cell e.s.d.'s are taken into account individually in the estimation of e.s.d.'s in distances, angles and torsion angles; correlations between e.s.d.'s in cell parameters are only used when they are defined by crystal symmetry. An approximate (isotropic) treatment of cell e.s.d.'s is used for estimating e.s.d.'s involving l.s. planes.
Refinement. Refinement of *F*^2^ against ALL reflections. The weighted *R*-factor *wR* and goodness of fit *S* are based on *F*^2^, conventional *R*-factors *R* are based on *F*, with *F* set to zero for negative *F*^2^. The threshold expression of *F*^2^ > σ(*F*^2^) is used only for calculating *R*-factors(gt) *etc*. and is not relevant to the choice of reflections for refinement. *R*-factors based on *F*^2^ are statistically about twice as large as those based on *F*, and *R*- factors based on ALL data will be even larger.

### Fractional atomic coordinates and isotropic or equivalent isotropic displacement parameters (Å^2^)

**Table:**

	*x*	*y*	*z*	*U*_iso_*/*U*_eq_	
Yb1	0.355312 (10)	0.07841 (3)	0.714967 (16)	0.01086 (9)	
S1	0.5000	0.0299 (3)	0.7500	0.0182 (4)	
C1	0.3390 (2)	0.0272 (7)	0.9554 (4)	0.0134 (10)	
C2	0.3697 (2)	0.2318 (7)	0.9654 (4)	0.0148 (10)	
C3	0.3764 (2)	0.3838 (7)	1.0402 (4)	0.0145 (10)	
C4	0.3492 (2)	0.4128 (7)	1.1340 (4)	0.0128 (10)	
C5	0.4168 (2)	0.4805 (8)	0.9077 (4)	0.0179 (11)	
H5	0.4371	0.5603	0.8664	0.022*	
N1	0.3942 (2)	0.2930 (6)	0.8817 (3)	0.0165 (9)	
N2	0.4065 (2)	0.5391 (6)	1.0009 (3)	0.0183 (10)	
H1	0.420 (3)	0.648 (6)	1.039 (4)	0.027*	
O1	0.32418 (19)	−0.0583 (5)	1.0318 (3)	0.0220 (9)	
O2	0.32955 (18)	−0.0546 (5)	0.8633 (3)	0.0192 (8)	
O3	0.33204 (18)	0.2591 (5)	1.1767 (3)	0.0219 (8)	
O4	0.34067 (19)	0.5917 (5)	1.1620 (3)	0.0219 (8)	
O5	0.45816 (18)	−0.0925 (5)	0.7997 (3)	0.0304 (10)	
O6	0.45308 (19)	0.1572 (6)	0.6710 (3)	0.0349 (10)	
O1W	0.24374 (18)	0.1038 (6)	0.6799 (3)	0.0250 (9)	
H2W	0.224 (2)	0.201 (6)	0.646 (4)	0.037*	
H1W	0.221 (3)	0.075 (8)	0.720 (4)	0.037*	

### Atomic displacement parameters (Å^2^)

**Table:**

	*U*^11^	*U*^22^	*U*^33^	*U*^12^	*U*^13^	*U*^23^
Yb1	0.01712 (13)	0.00743 (13)	0.00944 (13)	0.00016 (8)	0.00604 (9)	0.00022 (8)
S1	0.0156 (9)	0.0145 (9)	0.0250 (10)	0.000	0.0063 (8)	0.000
C1	0.017 (2)	0.010 (2)	0.012 (3)	0.0000 (19)	0.001 (2)	0.001 (2)
C2	0.021 (2)	0.012 (3)	0.011 (2)	−0.003 (2)	0.0040 (19)	0.000 (2)
C3	0.021 (3)	0.013 (2)	0.011 (2)	0.002 (2)	0.006 (2)	0.001 (2)
C4	0.017 (2)	0.010 (3)	0.010 (2)	−0.0018 (19)	0.002 (2)	−0.0017 (19)
C5	0.025 (3)	0.015 (3)	0.015 (3)	−0.005 (2)	0.008 (2)	0.003 (2)
N1	0.024 (2)	0.012 (2)	0.016 (2)	−0.0023 (17)	0.0082 (18)	−0.0010 (17)
N2	0.027 (2)	0.014 (2)	0.016 (2)	−0.0064 (19)	0.0084 (19)	−0.0018 (18)
O1	0.042 (2)	0.0151 (19)	0.0107 (19)	−0.0090 (16)	0.0095 (17)	−0.0001 (15)
O2	0.033 (2)	0.0155 (19)	0.0110 (18)	−0.0084 (15)	0.0090 (15)	−0.0058 (15)
O3	0.037 (2)	0.0144 (19)	0.0182 (18)	0.0042 (16)	0.0134 (16)	0.0061 (16)
O4	0.039 (2)	0.0103 (19)	0.018 (2)	0.0028 (16)	0.0105 (17)	−0.0009 (14)
O5	0.021 (2)	0.030 (2)	0.041 (3)	0.0019 (16)	0.0104 (18)	0.0155 (19)
O6	0.023 (2)	0.044 (2)	0.041 (3)	0.0071 (19)	0.0135 (19)	0.027 (2)
O1W	0.024 (2)	0.023 (2)	0.031 (2)	0.0066 (17)	0.0129 (18)	0.0083 (17)

### Geometric parameters (Å, °)

**Table:**

Yb1—O4^i^	2.264 (3)	C1—C2	1.481 (6)
Yb1—O1^ii^	2.272 (3)	C2—C3	1.367 (7)
Yb1—O1W	2.280 (4)	C2—N1	1.377 (6)
Yb1—O3^ii^	2.291 (3)	C3—N2	1.369 (6)
Yb1—O2	2.297 (3)	C3—C4	1.485 (7)
Yb1—O6	2.342 (4)	C4—O3	1.249 (6)
Yb1—O5	2.421 (4)	C4—O4	1.255 (5)
Yb1—N1	2.510 (4)	C5—N1	1.328 (6)
Yb1—S1	2.9798 (3)	C5—N2	1.334 (7)
S1—O5^iii^	1.464 (4)	C5—H5	0.9300
S1—O5	1.464 (4)	N2—H1	0.87 (5)
S1—O6^iii^	1.470 (4)	O1—Yb1^iv^	2.272 (3)
S1—O6	1.470 (4)	O3—Yb1^iv^	2.291 (3)
S1—Yb1^iii^	2.9798 (3)	O4—Yb1^v^	2.264 (3)
C1—O1	1.244 (6)	O1W—H2W	0.82 (2)
C1—O2	1.266 (6)	O1W—H1W	0.82 (6)
			
O4^i^—Yb1—O1^ii^	76.46 (12)	O6^iii^—S1—O6	110.8 (4)
O4^i^—Yb1—O1W	79.74 (14)	O5^iii^—S1—Yb1	135.09 (15)
O1^ii^—Yb1—O1W	78.99 (15)	O5—S1—Yb1	53.75 (14)
O4^i^—Yb1—O3^ii^	148.74 (13)	O6^iii^—S1—Yb1	120.86 (16)
O1^ii^—Yb1—O3^ii^	74.75 (12)	O6—S1—Yb1	50.65 (15)
O1W—Yb1—O3^ii^	83.05 (14)	O5^iii^—S1—Yb1^iii^	53.75 (14)
O4^i^—Yb1—O2	124.73 (12)	O5—S1—Yb1^iii^	135.09 (15)
O1^ii^—Yb1—O2	140.73 (13)	O6^iii^—S1—Yb1^iii^	50.65 (15)
O1W—Yb1—O2	74.07 (14)	O6—S1—Yb1^iii^	120.86 (16)
O3^ii^—Yb1—O2	74.10 (12)	Yb1—S1—Yb1^iii^	167.75 (7)
O4^i^—Yb1—O6	76.89 (14)	O1—C1—O2	122.7 (4)
O1^ii^—Yb1—O6	77.64 (14)	O1—C1—C2	122.6 (4)
O1W—Yb1—O6	150.11 (14)	O2—C1—C2	114.7 (4)
O3^ii^—Yb1—O6	108.21 (14)	C3—C2—N1	110.6 (4)
O2—Yb1—O6	135.20 (13)	C3—C2—C1	132.9 (5)
O4^i^—Yb1—O5	127.57 (13)	N1—C2—C1	116.5 (4)
O1^ii^—Yb1—O5	114.21 (14)	C2—C3—N2	104.5 (4)
O1W—Yb1—O5	150.85 (13)	C2—C3—C4	132.8 (4)
O3^ii^—Yb1—O5	76.28 (13)	N2—C3—C4	121.8 (4)
O2—Yb1—O5	80.58 (13)	O3—C4—O4	123.2 (5)
O6—Yb1—O5	58.02 (13)	O3—C4—C3	118.5 (4)
O4^i^—Yb1—N1	72.99 (13)	O4—C4—C3	118.1 (4)
O1^ii^—Yb1—N1	148.68 (12)	N1—C5—N2	111.0 (4)
O1W—Yb1—N1	101.95 (14)	N1—C5—H5	124.5
O3^ii^—Yb1—N1	136.57 (13)	N2—C5—H5	124.5
O2—Yb1—N1	66.31 (12)	C5—N1—C2	105.0 (4)
O6—Yb1—N1	88.79 (15)	C5—N1—Yb1	138.2 (3)
O5—Yb1—N1	80.25 (14)	C2—N1—Yb1	113.6 (3)
O4^i^—Yb1—S1	101.39 (10)	C5—N2—C3	109.0 (4)
O1^ii^—Yb1—S1	98.30 (10)	C5—N2—H1	130 (4)
O1W—Yb1—S1	176.78 (11)	C3—N2—H1	121 (4)
O3^ii^—Yb1—S1	94.59 (10)	C1—O1—Yb1^iv^	141.5 (3)
O2—Yb1—S1	107.44 (9)	C1—O2—Yb1	127.3 (3)
O6—Yb1—S1	29.03 (9)	C4—O3—Yb1^iv^	143.5 (3)
O5—Yb1—S1	29.18 (9)	C4—O4—Yb1^v^	164.1 (3)
N1—Yb1—S1	81.27 (10)	S1—O5—Yb1	97.06 (18)
O5^iii^—S1—O5	113.5 (3)	S1—O6—Yb1	100.32 (19)
O5^iii^—S1—O6^iii^	103.9 (2)	Yb1—O1W—H2W	121 (4)
O5—S1—O6^iii^	112.4 (2)	Yb1—O1W—H1W	128 (4)
O5^iii^—S1—O6	112.4 (2)	H2W—O1W—H1W	102 (3)
O5—S1—O6	103.9 (2)		

Symmetry codes: (i) *x*, −*y*+1, *z*−1/2; (ii) *x*, −*y*, *z*−1/2; (iii) −*x*+1, *y*, −*z*+3/2; (iv) *x*, −*y*, *z*+1/2; (v) *x*, −*y*+1, *z*+1/2.

### Hydrogen-bond geometry (Å, °)

**Table:**

*D*—H···*A*	*D*—H	H···*A*	*D*···*A*	*D*—H···*A*
N2—H1···O6^v^	0.87 (5)	2.09 (3)	2.925 (6)	161 (5)
O1W—H2W···O2^vi^	0.82 (2)	1.94 (3)	2.693 (5)	151 (5)
O1W—H1W···O3^vii^	0.82 (6)	2.24 (4)	2.896 (5)	138 (5)
O1W—H1W···O4^vii^	0.82 (6)	2.51 (6)	3.308 (5)	167 (5)
C5—H5···O5^viii^	0.93	2.52	3.347 (6)	149.

Symmetry codes: (v) *x*, −*y*+1, *z*+1/2; (vi) −*x*+1/2, *y*+1/2, −*z*+3/2; (vii) −*x*+1/2, −*y*+1/2, −*z*+2; (viii) *x*, *y*+1, *z*.
